# Challenges and perceptions of implementing mass testing, treatment and tracking in malaria control: a qualitative study in Pakro sub-district of Ghana

**DOI:** 10.1186/s12889-019-7037-1

**Published:** 2019-06-06

**Authors:** Ignatius Cheng Ndong, Daniel Okyere, Juliana Yartey Enos, Alfred Amambua-Ngwa, Corinne Simone C. Merle, Alexander Nyarko, Kwadwo Ansah Koram, Collins Stephan Ahorlu

**Affiliations:** 1 Department of Epidemiology, Noguchi Memorial Institute for Medical Research, College of Health Sciences, University of Ghana, Legon, Accra, Ghana; 2 Department of Biochemistry, Faculty of Science, Catholic University of Cameroon, Bamenda, Cameroon; 3 Medical Research Council Unit, The Gambia at London School of Hygiene and Tropical Medicine, Fajara, Gambia; 4 Special Programme for Research & Training in Tropical Diseases (TDR), World Health Organization, Geneva, Switzerland; 5 Department of Clinical Pathology, Noguchi Memorial Institute for Medical Research, College of health Sciences, University of Ghana, Accra, Ghana

**Keywords:** Malaria, Perceptions, Challenges, Test, Treat and track, Ghana

## Abstract

**Background:**

Malaria remains endemic in Ghana despite several interventions. Studies have demonstrated very high levels of asymptomatic malaria parasitaemia in both under-five and school-age children. Mass testing, treatment and tracking (MTTT) of malaria in communities is being proposed for implementation with the argument that it can reduce parasite load, amplify gains from the other control interventions and consequently lead to elimination. However, challenges associated with implementing MTTT such as feasibility, levels of coverage to be achieved for effectiveness, community perceptions and cost implications need to be clearly understood. This qualitative study was therefore conducted in an area with on-going MTTT to assess community and health workers’ perceptions about feasibility of scale-up and effectiveness to guide scale-up decisions.

**Methods:**

This qualitative study employed purposive sampling to select the study participants**.** Ten focus group discussions (FGDs) were conducted in seven communities; eight with community members (*n* = 80) and two with health workers (*n* = 14). In addition, two in-depth interviews (IDI) were conducted, one with a Physician Assistant and another with a Laboratory Technician at the health facility. All interviews were recorded, transcribed, translated and analyzed using QSR NVivo 12.

**Results:**

Both health workers and community members expressed positive perceptions about the feasibility of implementation and effectiveness of MTTT as an intervention that could reduce the burden of malaria in the community. MTTT implementation was perceived to have increased sensitisation about malaria, reduced the incidence of malaria, reduced household expenditure on malaria and alleviated the need to travel long distances for healthcare. Key challenges to implementation were doubts about the expertise of trained Community-Based Health Volunteers (CBHVs) to diagnose and treat malaria appropriately, side effects of Artemisinin-based Combination Therapies (ACTs) and misconceptions that CBHVs could infect children with epilepsy.

**Conclusion:**

The study demonstrated that MTTT was perceived to be effective in reducing malaria incidence and related hospital visits in participating communities. MTTT was deemed useful in breaking financial and geographical barriers to accessing healthcare. The interventions were feasible and acceptable to community members, despite observed challenges to implementation such as concerns about CBHVs’ knowledge and skills and reduced revenue from internally generated funds (IGF) of the health facility.

## Background

Presently, malaria is endemic in 91 countries down from 108 in 2000 [1]. Despite this remarkable progress, malaria remains a major health threat in sub-Saharan Africa. In 2016, an estimated 216 million cases of malaria were recorded with an estimated 445,000 deaths, with more than 90% of the deaths occurring in sub-Sahara Africa, 75% of which were children under five [2]. The success observed has been largely attributed to the introduction of nouvelle effective interventions such as ACTs, use of long lasting insecticidal nets (LLIN), indoor residual spraying (IRS), intermittent preventive treatment in infants and pregnant women (IPTc & IPTp), seasonal malaria chemoprevention (SMC) and test, treat and track (T3) approaches and continuous sensitisation [3–7].

A good number of interventions target people who are symptomatic or prevent those that are not infected from acquiring the malaria parasite using nets and other vector control measures. Though mass drug administration (MDA) was in used in the 1950s in malaria eradication programme, it has hardly been implemented in national malaria control programmes due to lack of knowledge on feasibility, drug efficacy and development of resistance to the drugs [8]. Recent reports from West Africa and South East Asia suggest that MDA is safe, well tolerated, feasible, and has the potential to achieved high population coverage and adherence if integrated into elimination programmes [3, 4, 8–10]. The MDA strategy adopted by the WHO for malaria is not yet part of the package for national malaria control programmes in endemic communities in West Africa.

Asymptomatic malaria is simply defined as malaria parasitaemia of any density devoid of fever or any acute symptoms in persons who have not recently received anti-malarials, though this may have significant health and social risks [11]. These neglected asymptomatic carriers which serve as reservoirs of the malaria parasite do not only sustain transmission in endemic areas but have been reported to be associated with chronic anaemia, maternal and neonatal morbidity and mortality and co-infections with invasive bacteria [4, 11, 12]. To improve efforts for early detection of new symptomatic cases at the community level, CBHV have been introduced, but the WHO guidelines for the T3 strategy do not include asymptomatic carriers [5]. Studies in Ghana have reported asymptomatic malaria parasitaemia carriage of 25% in children under-5 and above 40% in school-age children [12–15]. A longitudinal study in Coastal Ghana has demonstrated that linking IPTc with community-based management of malaria, delivered by trained CBHVs, could clear more than 90% of asymptomatic parasitaemia in children [12]. Though this study was done only in children under 5, the questions that led to the present study included: Could this approach or strategy be scaled-up to the entire population through MTTT? What are the bottlenecks to implementation of MTTT on a large scale? During MDA in the Greater Mekong Sub-region, high community engagement and population coverage was reported [3, 10, 16]. Data on MTTT interventions are required to inform relevant decision-making in Ghana and other malaria endemic communities in sub-Sahara Africa.

MTTT is increasingly being proposed for implementation with the argument that it will reduce parasite load and amplify gains from existing interventions such as use of LLIN, IRS, IPTp and IPTc which may consequently lead to elimination. Though challenges and benefits of implementing MDA in the fight against malaria are well documented in South East Asia [10, 17–20], the challenges associated with implementing MTTT in Ghana are unclear, including communities’ and health workers’ perceptions of MTTT. This study was therefore conducted to assess community members’ and health workers’ perceptions about MTTT.

## Methods

### Study design

This study adopted a narrative approach to qualitative research which allows participants to share their experiences on the MTTT interventions [21]. The approach was deemed suitable because the study aimed at gaining deeper insight into community perceptions about malaria MTTT implementation and related challenges.

### Study area

Pakro is one of five sub-districts in the Akwapim south district health directorate (DHD) in the Eastern region of Ghana [22]. The Akwapim south district lies within the semi-equatorial climatic region, and experiences two rainfall seasons in a year, with an average rainfall of 125 cm to 200 cm. The first rainy season begins from May to June with the heaviest rainfall in June, whilst the second rainy season occurs from September to October. According to the Ghana Statistical Service (GSS), the average household size in the Akwapim South district is about 4.0 whilst the average number of households per house or compound is estimated to be 1.6 [23]. The Pakro sub-district has an estimated population of 7889 and is bounded to the east by Akwapim North district; to the north by Ayensuano district; and to the west by Nsawam Adoagyiri Municipality. The sub-district is made up of 22 communities and has 3 healthcare facilities (1 Health Centre and 2 Community-based Health Planning Service (CHPS) compounds) [22]. Due to limited resources, free MTTT was implemented in seven communities, selected with the help of district and sub district authorities; Abease Newsite, Fante Town, Zongo (Adjenase/Kweitey), Piem/Odumsisi, Adesa, Sacchi/Tabankro and Odumtokuro. These communities had relatively higher population densities. The Pakro Health Centre is one of thirty sentinel sites for monitoring malaria prevalence in the country coordinated by the Noguchi Memorial Institute for Medical Research (NMIMR) of the University of Ghana. Malaria parasite positivity rate was estimated to be 45.7% in 2014, while anaemia among pregnant women at 36 weeks of gestation was 21% [22]. Malaria records the highest number of cases at the outpatient department of the Pakro health facility. Hence, this sub-district was purposively selected for the MTTT intervention. There are 18 trained health workers in the sub district – 1 Assistant Physician and 17 nurses. Fifteen of the nurses work in the two facilities within the selected communities. Seven of the nurses were involved in the consultation and treatment of patients in the outpatient department (OPD). LLINs had been distributed in the area a year before the implementation of MTTT. Fourteen Community-Based Health Volunteers (CBHVs) were specifically recruited and trained for MTTT on the use of RDT test kits, treatment following the treatment guidelines and follow-up, as well as reporting adverse events. These are community members without a health background.

### Selection of study participants

This qualitative study employed purposive sampling, a non-probabilistic sampling procedure. In purposive sampling technique, the researchers choose the sample based on who they think are appropriate for the study [24]. Purposive sampling technique is widely used in qualitative research and is appropriate to identify and select information-rich cases for the most effective use of limited resources [25]. In the study communities where MTTT was implemented we visited people who had benefitted from the programme and sampled participants from various houses for interview. Health workers in the health facilities were also purposively sampled and interviewed. This was done to provide a holistic perspective from community members and health workers. To be selected to participate in the FDG, one had to be a chief, an opinion leader, a family head or a woman (or guardian) of children under five. At the level of the health facility in sub-district, nurses involved in outpatient consultations were selected, while at the district level we selected participants from the malaria and nutrition units, and the administrative staff that were involved in coordination of the district health service.

### Sensitisation

To engage the communities, we collaborated with the Ghana Health Service (GHS) through the National Malaria Control Programme (NMCP) at the national, regional, district and sub-district levels. Community sensitisation was done through meetings/durbars with chiefs and opinion leaders [3, 12, 17]. We did not provide incentives to the participants above five years but all children under 5 were given chocolate cubes as an incentive. A few days to each intervention the participants were sensitised using a public address system. This entailed explaining why we were conducting MTTT, informing them on when the intervention will start and the importance of participating in the process, being treated when positive, the need to ensure that each family member completed the treatment, if positive, as well as reporting all side effects. When challenges such as misconceptions were reported, we run a sensitisation campaign to educate the population before continuing the programme. All these steps were taken to enable the community to develop an understanding and ownership of the project.

### Implementation of the MTTT programme

The MTTT Implementation team moved from house-to-house testing all participants with RDTs and treating those who were positive for malaria parasitaemia using ACTs [26]. To ensure that the entire population was reached, we used a community register established for this purpose. To reduce the effect of CBHVs covering long distances, we divided the study communities into catchment areas and each CBHV was assigned to cover a specific area. Where the catchment area was large, the population assigned to a CBHV was smaller. All those who were treated were followed up on days 1, 2, 3 and 7 to ensure compliance and monitored for adverse events. Between MTTT interventions the CBHVs conducted malaria tests using RDTs for all febrile cases at home, treated positive cases and referred non-malaria febrile cases to the Health Centre. All visits, surveys and interventions data of the participants were recorded in the community register. MTTT interventions were provided at no cost to the community.

### Data collection strategy

Focus group discussions (FGDs) and in-depth interviews (IDIs) were the main data collection strategies employed in this study with both men and women brought together during each FDG session. Ten FGDs were held in seven communities in the study site. Eight of the FGDs were held with community members whilst two were with various categories of health workers (administrators, nurses, disease control officer, health information officers, nutrition officer and data managers). One FGD was held in each of the seven communities except for Adjenase, which has a high Muslim population, where an additional FDG was held for the Muslims only, resulting in a total of 8 FGDs. Two IDIs were used to generate more in-depth information from individuals [24, 27]. With this approach, respondents have the opportunity to situate their own perceptions within the larger social context of MTTT and share the various challenges associated with its implementation.

All selected participants were reminded of the time and venue of the event a day before. In each community, a location was chosen in consultation with the participants, where all participants assembled for the FDG. The FDG for health workers took place at two locations – one at the Health Centre and another at the premises of the district health administration. At the end of the FDG, transportation was provided for all participants. We did not observe refusal to participate in the FDGs, rather some were eager to tell their story.

### Data collection tool

Semi-structured FGD and IDI guides were used to collect the qualitative data [28]. The topic guides contained themes such as health problems in the community, perceptions about malaria risk and incidence, malaria case management, cost of treatment, views about the MTTT programme, challenges to implementation and how to improve the programme. Following the training of data collectors, the collection tools were pre-tested in a community outside the study area. Findings from the pre-testing were used to refine the tool before the actual study.

### Data collection procedure

FGD participants were made to sit in a U-shaped arrangement with the moderator and note-taker seated in front of the participants. The FGDs were conducted by trained research assistants with experience in qualitative data collection. During the FGD, each participant was given the opportunity to respond to issues raised by the moderator for discussion. This was done to ensure that all participants had equal opportunity to actively participate in the discussions. Inductive probing was done on emerging new issues. Each FGD session took between 45 and 60 min to complete. Both FGD and IDI sessions were recorded with the permission of participants. Audio-recordings were re-played to participants as required in member checking, a quality control measure in qualitative research [29]. Detailed field notes were taken during the discussions to complement the audio recordings.

### Data transcription and analysis

At the end of data collection, all voice recordings were translated from the local language to English during transcription. The transcripts were reviewed by comparing the translated versions with the original voice recordings. The researchers read through the transcripts noting the emerging issues. These were transformed into a codebook. The transcripts were imported into NVivo 12 software. The codebook was also imported into the software as nodes. Coding of each transcript was conducted within NVivo based on the code definition developed by the team in the codebook. Double coding was done and the code comparison query performed as quality strategy in data analysis [30]. Thematic content analysis [24] was used in this study. The results are presented in narratives according to the themes that emerged during analysis.

## Results

### Background characteristics of participants

Ten focus group discussions (FGDs) were conducted in seven communities; eight with community members (*N* = 80, with number of participants per group ranging from 6 to 13) and two with health workers (*N* = 14, 7 participants per group). In addition, two in-depth interviews (IDIs) were conducted with the physician Assistant and the Laboratory Technician.

### Overview of participant views

In generally, participants believed that the MTTT interventions had been effective in reducing the burden of malaria in the participating communities. They perceived the benefits of MTTT as follows: i) reducing the incidence of malaria in children, ii) increased sensitisation about malaria, iii) reduced frequency of hospital attendance and time-saving for productivity, iv) reduced household expenditure for malaria treatment, v) providing prompt and timely access to management of malaria at home, vi) alleviating the need to travel long distances to neighbouring communities and facilities for healthcare, and on the flip side, vii) a stated concern of health workers about revenue loss to the facility as part of their IGF as a result of reduced clinic visits.

i) Reduced incidence of malaria in children

Most of the participants felt that there had been a decline in malaria incidence in the community since the introduction of MTTT interventions coupled with the community-based management of malaria. Both community participants and health workers were of the opinion that the implementation of MTTT did provide a lot of benefit to the community. Quotes:

*“Before the pricking (MTTT), you realise that most of the children fall ill a lot. They vomit and have high temperatures but now the number of times they fall ill has reduced”* (P3, Piem, FGD).

*“There is a reduction in the sickness [malaria]. Your work has really helped us. We do not need to rush to the clinic anymore”* (P4, Fante Town, FGD).

*“My child usually gets sick a lot and vomits too. But since the pricking (MTTT) started, he is okay now*” (P6, Abease, FGD).

*“Eerm, truth be told, malaria has worried us a lot in this area, but it has reduced since your team [MTTT] came. Also we know the volunteers (CBHVs) have some drugs at home, so we go to them when we are sick”* (P5, Adesa Nsuablaso, FGD).

ii) *Reduction in the frequency of hospital attendance*

Participants in this study were of the view that the MTTT programme had reduced the frequency of hospital attendance for malaria episodes and increased awareness of the community about malaria prevention and management. To community participants, CBHVs often provide education about malaria and bed-net use as part of the MTTT intervention at home. Participants were of the view that the MTTT has contributed to improving the health and wellbeing of families in the community. This was indicated by the perceived reduction in malaria-related hospital visits or drugs store visits as illustrated in the narrative below:

*“I agree that malaria has been a very serious problem for us and we used to go to the hospital all the time. Due to your intervention (MTTT), my grandchild and I have not visited the hospital this year”* (P10, Abease, FGD).

*“My kids are young and mostly suffer from malaria. The hospital gave me the nick name “malaria company” due to the frequent attendance to the clinic. I can see a change now, it has reduced because of the MTTT intervention. I no longer buy malaria drugs from the drug store”* (P1, Adjenase, FGD, NM).

*“I can say even without the statistics that it is yielding positive results. This is because it has drastically brought down our attendance and number of malaria cases in the clinic”* (Health worker, IDI, Pakro).

iii) *Saving time for productivity*

According to respondents, the MTTT programme was impactful in improving the economic status of the family through savings of productive time for adults. Community members felt that visiting a health facility for malaria care required the individual to spend some at the facility. Also, caretakers had to accompany patients to the health facility for care. This is perceived to take the community members away from their work. Thus, attending to patients at home is perceived to be time-saving. Caretakers are able to use the time that would have been spent at the health facility to engage in other productive activities to earn more income for the household. The following illustrate these points:

*“[…] it has saved us time. When you are tested and treated at home it saves you the days or weeks you will have to spend at the clinic but when you are treated at home you use that time to do business that will bring more income*” (P11, Abease, FGD).

*“There is a saying that ‘time is money’. The time I will spend at the hospital can be saved for something else to make more money for my family. Transportation cost is also saved”* (P8, Adjenase, FGD).

Implementation of MTTT interventions in communities that lacked a health facility was welcomed as it prevented them from walking long distances to a health facility to receive care for malaria. The presence of the CBHVs in the community and the availability of the free MTTT services seem to have increased the willingness to seek care compared to when they had to go long distances to consult and pay out-of-pocket for treatment as revealed by this quote:

*“We do not have a health facility in this community, so when one is ill of malaria, you have to walk a long distance to the health facility located in the next village. Now we don’t have to walk again, you just go to the volunteer (CBHVs) for the test and treatment”* (P10, Piem, FGD)

iv) *Early detection of malaria, improved awareness and health seeking behaviour*

Respondents believed that the programme had led to early detection of malaria cases and treatment in the study communities. The programme had also increased awareness in the communities that an individual can carry the malaria parasite without being ill. The engagement of CBHVs for the MTTT interventions is thought to have helped in reducing the severity of malaria in the communities. Participants generally felt that the MTTT programme had reduced the burden of malaria and also reduced the tendency for self-medication through drug stores. Quotes:

*“We are impressed with your work (MTTT). I usually don’t go to the hospital, so I did not know that I had the malaria in my blood until you came to test me. Though I was not sick, I tested positive and they treated me. This has helped in reducing malaria in the community”* (P8, Odumtokro, FGD).

*“Malaria is still there but not as severe as it used to be without your work (MTTT)”* (P7, Abease, FGD).

v) *Prompt and timely access to malaria case management at home*

Following implementation of MTTT, study participants were of the opinion that treatment for malaria had become more readily accessible in the community and at home through the CBHVs, leading to a reduction in malaria incidence in the communities. The availability of health facilities in the community or neighbouring communities made it possible for people with malaria to seek prompt and timely health care. Though the participants generally understood the need to seek early treatment for malaria at health facilities, they indicated that prior to implementation of MTTT; most of the community members treated malaria with herbs at home and only visited health facilities when the home remedy approach failed, or when the illness became severe or complicated. These observations demonstrate that MTTT addressed some of the barriers to seeking healthcare such as distance and cost. Before initiation of the MTTT project, there were no operational CBHV activities in the sub district, though some CBHVs had been trained in the past.

*“Before the start of the programme (MTTT) in the community, we first treated malaria with local herbs and if it persisted then, we reported to the clinic. But now, we have access to the drugs from the community volunteers and therefore consult them first”* (P6, Sachi/Tabankro, FGD)

*“Looking at the fact that the hospital is far from this community and the cost of treatment is high, the first option was for us to rely on herbs. But with your (MTTT) coming, it has changed. We go to the volunteers first, they test and treat us for free”* (P7, Piem, FGD).

vi) *Reduced malaria-related healthcare cost*

Cost of managing malaria was also identified by community members as a barrier to seeking care at health facilities. According to participants, it cost between GH¢28 (US$5.9) to GH¢200 (US$42.0) to treat malaria in the community. This cost covers transportation to the health facilities in some instances, food and hospital fees (consultation, lab and drugs). With the advent of MTTT complemented by community-based management of malaria, the participants appreciated the activities of the CBHVs who provided free testing and treatment services at home, which contributed to alleviating the cost of seeking treatment. Participants who used the services of CBHVs in the community especially in between MTTT interventions were of the opinion that it was helpful to them. The following illustrate these points:

“*My child recently fell sick. The hospital confirmed it was malaria and was treated. I paid for the drug. It cost me GH¢20.00 (*US$4.2)*. Motor transport also cost me GH¢8.00 (*US$1.7)*, so a total of GH¢28.00* (US$5.9)*”* (P1, Sachi Tabankro FGD).

*“My grandchild will not be okay if he does not take about 2 or 3 infusions. Just within last month I spent about GH₵80.00 (*US$16.8) *and last week another ₵60.00 (*US$12.6)*. But just 3 days ago I went to the volunteer and it was free. This helped me”* (P2, Abease, FGD).

The perceived benefits of MTTT goes beyond the services being free but this enabled the families to save money which can be used to take care of other family’s needs as revealed by the following quotes.

*“I have realized that spending about GH₵60.00 (US*$ *12.6) to GH₵100.00 (US*$*21.0) at the hospital is now a thing of the past. All the money is saved in our pockets”* (P7, Piem, FGD)

*“We save the money meant for hospital, because the child can be treated by the volunteer. So the money for transportation and drugs can be used for other things. They come to our houses and treat us”* (P4, Abease, FGD).

vii) *Impact of MTTT on the health system*

Health workers corroborated that there was a decline in febrile cases in recent times as compared to the last two years. They felt that fewer febrile cases were reporting at health facilities since the inception of the MTTT programme. However, they were of the opinion that the lost revenue from averted febrile cases was adversely affecting the resource stream of the health facility. The health system in Ghana is partly funded by internally generated funds (IGF) from out-of-pocket payments and payments through the National Health Insurance Scheme (NHIS) at the level of each facility. In rural areas, a bulk of the revenue comes from outpatient consultations especially from febrile cases. Malaria is one of the diseases that are covered by the National Health Insurance Scheme (NHIS). Patients under this scheme have part of their bills paid by the scheme while those not under the scheme pay for services out-of-pocket [12, 26, 31]. The following quote illustrates this:

*“Within the first 2 months after commencement of the programme, our revenue dropped because most of our cases were treated at home, so they were not coming to our facility. It affected insured and non-insured attendance as well. It really affected our revenue*” (P6, Health worker, FGD).

The number of febrile patients being tested for malaria-related illnesses seems to constitute an important source of revenue for the health facility. This is demonstrated by a decrease in the number of febrile cases in the health facility being perceived to be directly related to the reduction in the revenue generated by the facility over the period the MTTT interventions were implemented in the area. This seems to reflect a mixed blessing – on the one hand it represents an advantage to the community that the level of malaria prevalence will decline and improve the health of the community. On the other hand, the health facility is constrained and risks collapsing if alternative funding is not sought to support its services, which hitherto was supported principally by revenue generated from malaria-related consultations. Most of the febrile illnesses being handled at the community level by CBHVs would have come to the health facility. This means that only the non-malaria cases report to the facility. This is possible given that when the asymptomatic malaria pressure is reduced the risk of some invasive bacterial infections consequently reduces [4]. This is demonstrated by the following quotes:

*“Your project (MTTT) is doing very well, the community members are happy […] hhhhmmm […] but you have ‘finished us‘[…] you have collapsed the health facility. You have cleared the parasite from the community and the people are fine but we no longer have patients […] the health system will collapse. Even at the district level, they (district authorities) asked me what is happening to our attendance and I said it was the effect of your project (MTTT)”* (Health worker, Pakro facility, IDI).

*“You know that the hospital uses the internal revenue generated from malaria consultations to handle other diseases. So if malaria is to be handled this way then the government has to seek alternative funding for the health system […]”* (Health worker, Pakro facility, FGD)

### Tracking of malaria cases and treatment

Participants indicated that the MTTT programme had contributed to tracking malaria cases in the community. They were of the view that CBHVs effectively conducted follow-up visits to community members at home for testing. The fact that this was done at various times of the day ensured that every member of the household is captured by the intervention. The participants felt that the community register developed for this purpose was very helpful. Through these efforts, all members in the catchment area eventually got tested and treated when the test was positive. The following illustrate these points:

*“I am impressed with the fact that anyone who is not available when the volunteers come around to test eventually gets tested. They come around many times until we are tested. They have all our names. So they follow the list”* (P11, Adjenase, FGD-M).

*“They come at a time when everyone has returned from the farm and they have time for you. So everybody gets the opportunity to be tested and treated”* (P9, Fante Town, FGD).

Following-up treated cases was greatly appreciated by the participants who felt that they were being given attention. Also conducting subsequent rounds of MTTT gave the community members some degree of assurance as it was perceived as a confirmation whether someone was still carrying the parasite following the previous malaria episode or was negative.

*“When they treat you for malaria, they will come back to check whether you have taken the treatment. This is very important because you know that somebody cares about you”* (P11, Adjenase, FGD-M)

*“I remember my son was vomiting very severely and I did not know what to do. By the grace of God, the people who were pricking (MTTT) came to my aid, tested and treated him for free. The next time they came he was negative”* (P3, Adjenase, FGD-NM).

*“I have a grandchild who used to have malaria almost every two weeks. But recently the test showed that he was negative”* (P1, Adesa Nsuablaso).

### Challenges experienced during MTTT implementation

It was generally acknowledged that some people did not participate in the MTTT activities as a result of misconceptions and rumours spread in the community. One reason for their refusal to participate was the perception that health workers were infecting people, particularly children, with *“asram”* (local name for epilepsy). They believed that epilepsy is introduced into the blood of the person through the needle prick and that some of the volunteers were spiritualists. Though very few people felt this way, it was feared that this could contaminate the rest of the community. For this reason, we conducted a sensitisation campaign before each intervention. The following quotes support these claims by FGD participants:

*“I don’t have any problem with them (CBHVs), but I have heard rumors that one volunteer can infect children with “asram” (Epilepsy) through the needle prick*” (P4, Adjenase, FGD, NM).

Interestingly, those who tested negative for malaria in first rounds of MTTT felt that they were not susceptible to malaria. Though few, this group of participants did not see the need to be tested during subsequent interventions. To them it was inappropriate to receive a needle prick only to be told that the test is negative.

*“People sometimes refuse to be tested because anytime the test is done it is negative. So they do not want to be pricked to have pain and be told they are negative”* (P10, Fante Town)

Another barrier to MTTT was the inconvenience of taking the medicine. Some community members did not like the medicine because they experienced side effects such as stomach upset, dizziness or headache after taking the medicine. The CBHVs had to do a follow-up to encourage the participants on treatment to take the drug. Also some felt that though they carried the parasite, they do not need to take medicine and suffer from the side effects when they did not have the symptoms of the disease. The participants in this situation were advised to eat well before taking the medication. In some cases, the participants were willing to eat and take the drugs. However, in a few cases it was realised that some of the participants had nothing to eat at that moment. The project team sometimes went out of their way to provide food to enable the participants take their medication.

*“I know the disease is in my blood but the medicine they gave me caused stomach upset. So, I stopped taking it and told myself that I won’t participate again” (P4, Adesa Nsuablaso).*

The key informants acknowledged that many community members do not like taking Artesunate Amodiaquine because they complain of dizziness and weakness after taking the drug. It was therefore recommended that Artemether Lumefantrine- should be used during MTTT for such cases.

*“When we give Artesunate Amodiaquine even in the clinic, they end up returning to the clinic with severe weakness and those things. We have resorted to using the Artemether Lumefantrine as our first line. So, majority of the people are confident in the Artemether Lumefantrine”* (Health worker, IDI, Pakro).

### Lack of trust in the volunteers

The belief among community members was that CBHVs participating in the MTTT were not trained health workers and therefore lacked the expertise in diagnosing and treating people with malaria. This attitude, however, changed over time.

*“At the beginning, some people refuse to test because they believe the volunteers are from the same community without any training on health”* (P4, Fante Town, FGD)

### Community recommendation to improve the MTTT implementation

An important outcome of the FGDs was that the participants took ownership of the study and made some suggestions that could enhance the implementation of the MTTT programme in the future. They suggested that testing be conducted preferably in the morning and that multivitamins be added to the malaria treatment regimen provided by the MTTT programme. These recommendations are mentioned below:

*“The needle is painful. So they should not come pricking us in the afternoon. It is much more painful to prick in the afternoon. It is better in the morning”* (P7, Abease, FGD).

*“We become weak after swallowing the drug, so if you can add some multivitamin to the treatment, it will help us”* (P4, Abease, FGD).

Furthermore, the participants felt that the 4 months interval between the MTTT interventions was too long. They felt that the intervention could be more effective in reducing malaria incidence if the interval between two interventions was reduced to 2 months as illustrated:

*“I think you should reduce the length of time that you come to test us from every 4 months to every 2 months”* (P8, Adesa Nsuablaso, FGD).

*“The 4 months interval period is too long. When you stay too long before you come back we will have malaria again. But if you come after a short time we will not be ill of malaria again”* (P1 Sachi Tabankro, FGD).

## Discussion

This study attempts to assess community perceptions about the MTTT interventions that was undertaken in the Pakro sub-district of Ghana. Malaria is believed to pose a major health burden in the study communities, which was hitherto managed using self-medication, herbs and care seeking from the health facility when the first two options yielded no positive results, until the initiation of the MTTT programme. Implementing MTTT in the participating communities resulted in a decline in malaria incidence corroborating recent reports in Zambia, Kenya, Liberia and The Gambia [4, 9, 32–34] and increased malaria awareness, despite challenges such as lack of confidence in the expertise of CBHVs as well as decline in revenue witnessed by the health facilities in the catchment area. The perception of a decline in malaria incidence by the participants confirmed the findings of the quantitative component of the study *(forthcoming).* The findings clearly demonstrate that implementing MTTT complemented by community management of malaria through CBHVs facilitated prompt and timely access to malaria diagnoses and treatment and decreased the prevalence of febrile illnesses [4, 9, 10, 35], increased population coverage, freed up time for productivity and enabled families to save money from managing malaria [12, 36, 37].

The community acceptance of the MTTT approach to dealing with asymptomatic malaria parasitaemia provides a potential opportunity for scaling up the interventions, thus corroborating the findings from other parts of Africa and from MDA interventions in the Greater Mekong Sub-regions [3, 4, 9, 17, 20, 21, 38]. House-to-house implementation of MTTT seems to have contributed to the success of this strategy. The biggest challenge with the approach is the need to cover long distances as was reported by Peto et al. [17]. As explained earlier, this challenge was addressed in this study partly by dividing the study communities into catchment areas and each CBHV was assigned a specific area with a target population.

Similar to other findings, the CBHVs were dedicated to tracking participants to be tested as well as following-up those treated to ensure that they completed their treatment [32, 35, 39, 40]. This observation is commendable as it is an essential component of MTTT and further corroborates similar findings from the implementation of MDA in the Greater Mekong Sub-region [4, 8, 17, 41, 42]. Though the test, treat and track (T3) strategy is recommended by WHO [5], its present focus is on patients attending hospital for malaria-related illnesses but not asymptomatic cases at the community level. Expanding the T3 strategy to cover asymptomatic individuals across endemic communities could strengthen the malaria elimination efforts in line with the global agenda [43]. An earlier study in Ghana found that less than half (48.3%) of patients who were asked to return for review in the clinical setting did return [44]. Hence, tracking of malaria cases may not be effective if left to the patient alone. However, it could be improved upon if CBHVs are involved. This has the potential to propel to full implementation of an MTTT agenda and lead to a reduction in the burden of malaria in Ghana and could be expanded to other geographical regions in Africa. Due to continuous monitoring and replenishing of ACTs, RDTs and other consumables, stock-outs were not observed in the course of the study [33, 44].

Despite the positive perceptions of the intervention such as reducing malaria incidence and improving access to care, some challenges were observed and reported during the implementation. The notion that CBHVs were not trained and lack expertise to provide MTTT interventions has the potential to undermine the programme objectives in the community, corroborating earlier studies [32, 33, 45]. This challenge was addressed through continuous community sensitisation during the course of the study. The CBHVs for this project were selected from already existing pool of CBHVs identified by the health facility and trained for the MTTT. The perception that CBHVs could infect people with epilepsy is not only limited to Ghana. This has been observed in Southern Zambia where CBHVs were believed to be ritualists who could use the blood of children for satanic purposes and infect people with HIV [33].

Another challenge observed in this study was that patients were not completing treatment because of the belief that they were not sick, as well as the unpleasant feeling after taking the medication. These unpleasant feelings were reported to be common for Artesunate Amodiaquine (AA) [7, 12, 38, 46]. It has been reported that the feeling of dizziness and weakness after ACTs is often due to people taking the medication without eating or on an empty stomach [47]. These observations emphasized the need for continuous sensitisation of the population on the importance of eating well before taking medication [17, 41]. This could often be ‘easier said than done’ as some families may not be able to provide the meal at such moments. This notwithstanding, it was generally the view of both the community members and health workers that AL was a preferred antimalarial medication than AA. To ensure compliance to treatment by patients, it would be important to give AL to patients as it is believed to have less side-effects than AA. It must be noted that non-compliance with antimalarial treatment could lead to the emergence of drug resistance [48, 49]. As suggested, it will be useful to include some multivitamins in the intervention packages especially for children. Community engagement is usually intended for a project to be responsive, adaptive and able to adjust the project implementation to adopt recommendations [16, 17, 41]. However, in the context of the current study, all the recommendations were not immediately incorporated into the implementation process. However, it informed the discussions on the next phase of the project.

Even though, the current MTTT study implemented a four-month interval between interventions, community members desired a more frequent test, treat and tracking system. They recommended that the interval should be reduced to two months or less. This suggestion is important as implementing MTTT at short intervals could reduce the chances of re-establishment of the parasite pool in a particular community. To some extent this demonstrates communities’ ownership of MTTT. However, it is important to take into consideration the drug safety regulations and other logistic implications when considering the length of time between interventions. Despite the fact that some participants perceived the testing as painful, overall, the participants perceived the strategy as helpful in overcoming the burden of malaria [33].

Health workers generally perceived that though the intervention reduced malaria incidence, it adversely affected the revenue generated [internally generated fund (IGF)] by the health facilities operating in the catchment area of the project. This decline in revenue was perceived to be a consequence of a decrease in the number of febrile cases seeking care for malaria-related illnesses at the health facilities [4]. Since malaria remains one of the leading causes of morbidity and mortality in health facilities in endemic areas [50], implementing MTTT interventions could result in a continuous decline in revenue for health facilities. Malaria treatment is paid for partly out-of-pocket or by the NHIS for registered members. Therefore, a drastic reduction in the number of malaria cases could lead to a decrease in IGF from the scheme, which is a cost recovery system and not a profit-making system [26, 31, 51]. Thus, the MTTT interventions may be perceived to be having a negative effect on the revenue generation for the health facility. However, it is worth noting that these perceptions reflect the views of the health workers interviewed and not necessarily those of the district health management team. Broader stakeholder consultations are therefore needed to reflect on the veracity of the claim and explore alternative funding mechanisms for the health system, if the perceived revenue decline is real. This could curtail potential conflict among health workers contemplating the need to eliminate malaria and improve the health and wellbeing of the population through effective interventions such as MTTT in endemic communities and concerns about protecting the revenue generation capacity of the health facilities, and by extension, the health system.

### Limitations of the study

One of the limitations of this study is that we did not interview decision makers beyond the district level, as they might have provided a broader perspective on the implication of the findings of this study. Another limitation was that population movements in and out of the study area which could have impacted the results obtained were not monitored. Furthermore, concerning the adverse events reported by the participants, we did not attempt to differentiate what was conflated with their pure perceptions. This could also have impacted the findings.

## Conclusion

The findings of this study demonstrate that MTTT interventions are perceived to be effective in reducing malaria incidence and related hospital visits in participating communities. The interventions are feasible and acceptable to community members, despite observed challenges to implementation such as concerns about CBHVs’ knowledge and skills and reduced revenue from IGF. MTTT is also deemed to be useful in breaking financial and geographical barriers to accessing healthcare, as interventions are delivered free in the community by CBHVs, thus, eliminating the need for transportation and out-of-pocket payments.

However, concerns about declining revenue from IGF due to decreasing malaria cases at the health facility as a result of MTTT implementation need to be explored further. If these concerns are proven to be real, mechanisms must be established to ensure sustainable financing of health facilities that are not dependent on the revenue generated from consultations for endemic diseases such as malaria. Hence, in contemplating the scale-up of MTTT in malaria endemic communities, measures should be put in place to assure sustainable financing of health systems.

## Data Availability

The data analysis is available in the Department of Epidemiology, Noguchi Memorial Institute for Medical Research and can be made available upon reasonable request.

## Abbreviations

AA: Artesunate Amodiaquine
ACT: Artemisinin-based Combination Therapy
AL: Artemether Lumefantrine
CBHV: Community-Based Health Volunteer
CHPS: Community-based Health Planning and Services
ERC: Ethics Review Committee
FGD: Focus group discussion
GHS: Ghana Health Service
GSS: Ghana Statistical Service
IGF: Internally generated funds
IPTc: Intermittent preventive treatment in children
IPTp: Intermittent preventive treatment in pregnant women
IRS: Indoor residual spraying
LLIN: Long lasting insecticidal nets
MTTT: Mass Testing, Treatment and Tracking
NMCP: National malaria control programme
T3: Test, Treat, Track
WHO: World Health Organisation
